# 1,4-Bis[(2-ethyl-1*H*-benzimidazol-1-yl)meth­yl]benzene

**DOI:** 10.1107/S1600536811025402

**Published:** 2011-07-02

**Authors:** Qiang Wang, Xing Zhao, Haipeng Lou, Mengyan Wang

**Affiliations:** a The Department of Physics–Chemistry, Henan Polytechnic University, Jiao Zuo, 454000, People’s Republic of China

## Abstract

In the title mol­ecule, C_26_H_26_N_4_, the central benzene ring forms dihedral angles of 89.9 (2) and 85.4 (2)° with the two benzimidazole rings.

## Related literature

The title compound is a precursor of *N*,N*N*′-benzimidazolium ionic liquids (ILs). For background to the use of ILs as solvents or ligands in the synthesis of new metal-organic frameworks (MOFs), see: Fei *et al.* (2006 ▶); Wang *et al.* (2009 ▶); Xu *et al.* (2009 ▶). For properties of metal-containing ILs, see: Lee *et al.* (2004 ▶); Sasaki *et al.* (2005 ▶); Wang *et al.* (2009 ▶). For details of the synthesis, see Rajakannu *et al.* (2011 ▶).
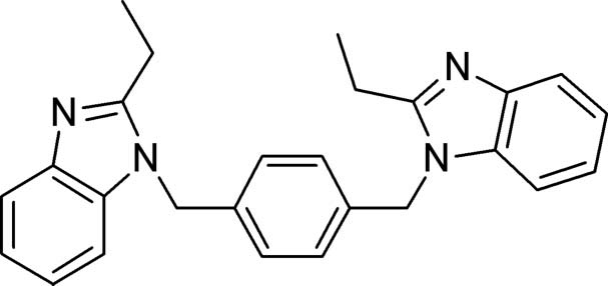


## Experimental

### 

#### Crystal data

C_26_H_26_N_4_
*M* *_r_* = 394.51
Orthorhombic, 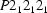

*a* = 10.300 (2) Å
*b* = 11.437 (2) Å
*c* = 17.809 (4) Å
*V* = 2098.0 (7) Å^3^
*Z* = 4
Mo *K*α radiation
μ = 0.08 mm^−1^
*T* = 298 K
0.20 × 0.16 × 0.15 mm

#### Data collection

Bruker SMART APEX diffractometer
Absorption correction: multi-scan (*SADABS*; Sheldrick, 2003 ▶) *T*_min_ = 0.986, *T*_max_ = 0.989
16782 measured reflections
2829 independent reflections
2488 reflections with *I* > 2σ(*I*)
*R*_int_ = 0.053

#### Refinement

*R*[*F* ^2^ > 2σ(*F* ^2^)] = 0.076
*wR*(*F* ^2^) = 0.153
*S* = 1.04
2829 reflections
273 parameters
H-atom parameters constrained
Δρ_max_ = 0.13 e Å^−3^
Δρ_min_ = −0.19 e Å^−3^

### 

Data collection: *APEX2* (Bruker, 2003 ▶); cell refinement: *SAINT* (Bruker, 2001 ▶); data reduction: *SAINT*; program(s) used to solve structure: *SHELXS97* (Sheldrick, 2008 ▶); program(s) used to refine structure: *SHELXL97* (Sheldrick, 2008 ▶); molecular graphics: *SHELXTL* (Sheldrick, 2008 ▶); software used to prepare material for publication: *SHELXTL*.

## Supplementary Material

Crystal structure: contains datablock(s) I, global. DOI: 10.1107/S1600536811025402/ng5188sup1.cif

Structure factors: contains datablock(s) I. DOI: 10.1107/S1600536811025402/ng5188Isup2.hkl

Supplementary material file. DOI: 10.1107/S1600536811025402/ng5188Isup3.cml

Additional supplementary materials:  crystallographic information; 3D view; checkCIF report

## Figures and Tables

**Fig. 1.**
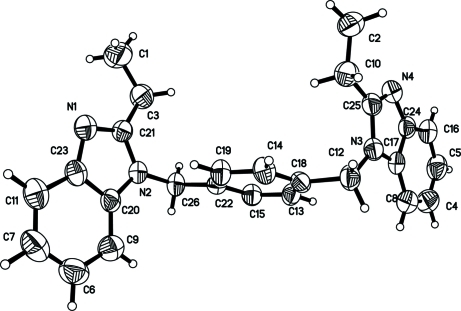
The molecular structure of the title molecule, with the atom-numbering scheme. Displacement ellipsoids are drawn at the 30% probability level.

## supplementary crystallographic information

### Comment

Ionic liquids (ILs) have attracted great interest as solvents or ligands in the synthesis of new metal-organic frameworks (MOFs)(Fei *et al.*,2006; Wang *et al.*, 2009; Xu *et al.*, 2009). By incorporating metal ions to the ILs, metal-containing ILs can be formed. the presence of metal ions in ILs provided many additional properties such as color, geometry, and magnetism (Lee *et al.*, 2004; Sasaki *et al.*, 2005; Wang *et al.*, 2009). As a part of our program devoted to the new applications of the ILs ligands in coordination polymer, we report herein the crystal structure of the title compound as the precursor of the N,N'-benzimidazolium ILs.

The molecule structure of title compound was shown in the Fig.1, all bond lengths and angles are in normal range. In the crystal structure, the central benzene ring forms dihedral angles of 88.9 (0)° and 84.4 (2)° with the two benzimidazole rings, respectively. The N2 atom are nearly co-planar with benzene ring by 0.055 (4) Å derivation. The crystal packing is stabilized by van der Waals forces.

### Experimental

(type here to add preparation details)

### Refinement

H atoms were located in a difference map and treated as riding with C—H = 0.96 Å for methyl groups and C—H = 0.93 Å for all other H atoms with *U*_iso_ (H) = 1.2 *U*_eq_ (aromatic, methylene) or *U*_iso_(H) = 1.5 *U*_eq_ (methyl).

### Crystal data

**Table:**

C_26_H_26_N_4_	*F*(000) = 840
*M**_r_* = 394.51	*D*_x_ = 1.249 Mg m^−^^3^
Orthorhombic, *P*2_1_2_1_2_1_	Mo *K*α radiation, λ = 0.71073 Å
*a* = 10.300 (2) Å	θ = 2.1–25.0°
*b* = 11.437 (2) Å	µ = 0.08 mm^−^^1^
*c* = 17.809 (4) Å	*T* = 298 K
*V* = 2098.0 (7) Å^3^	Block, colorless
*Z* = 4	0.20 × 0.16 × 0.15 mm

### Data collection

**Table:**

Bruker SMART APEX diffractometer	2829 independent reflections
Radiation source: fine-focus sealed tube	2488 reflections with *I* > 2σ(*I*)
graphite	*R*_int_ = 0.053
φ and ω scans	θ_max_ = 27.9°, θ_min_ = 2.1°
Absorption correction: multi-scan (*SADABS*; Sheldrick, 2003)	*h* = −13→11
*T*_min_ = 0.986, *T*_max_ = 0.989	*k* = −13→15
16782 measured reflections	*l* = −23→23

### Refinement

**Table:**

Refinement on *F*^2^	Primary atom site location: structure-invariant direct methods
Least-squares matrix: full	Secondary atom site location: difference Fourier map
*R*[*F*^2^ > 2σ(*F*^2^)] = 0.076	Hydrogen site location: inferred from neighbouring sites
*wR*(*F*^2^) = 0.153	H-atom parameters constrained
*S* = 1.04	*w* = 1/[σ^2^(*F*_o_^2^) + (0.0508*P*)^2^ + 1.*P*] where *P* = (*F*_o_^2^ + 2*F*_c_^2^)/3
2829 reflections	(Δ/σ)_max_ = 0.002
273 parameters	Δρ_max_ = 0.13 e Å^−^^3^
0 restraints	Δρ_min_ = −0.19 e Å^−^^3^

### Special details

**Table:**

Geometry. All e.s.d.'s (except the e.s.d. in the dihedral angle between two l.s. planes) are estimated using the full covariance matrix. The cell e.s.d.'s are taken into account individually in the estimation of e.s.d.'s in distances, angles and torsion angles; correlations between e.s.d.'s in cell parameters are only used when they are defined by crystal symmetry. An approximate (isotropic) treatment of cell e.s.d.'s is used for estimating e.s.d.'s involving l.s. planes.
Refinement. Refinement of *F*^2^ against ALL reflections. The weighted *R*-factor *wR* and goodness of fit *S* are based on *F*^2^, conventional *R*-factors *R* are based on *F*, with *F* set to zero for negative *F*^2^. The threshold expression of *F*^2^ > σ(*F*^2^) is used only for calculating *R*-factors(gt) *etc*. and is not relevant to the choice of reflections for refinement. *R*-factors based on *F*^2^ are statistically about twice as large as those based on *F*, and *R*- factors based on ALL data will be even larger.

### Fractional atomic coordinates and isotropic or equivalent isotropic displacement parameters (Å^2^)

**Table:**

	*x*	*y*	*z*	*U*_iso_*/*U*_eq_	
C1	0.7649 (6)	0.2700 (5)	0.3958 (3)	0.0931 (19)	
H1A	0.7571	0.1868	0.4012	0.140*	
H1B	0.7173	0.3081	0.4351	0.140*	
H1C	0.8547	0.2919	0.3988	0.140*	
C2	0.8228 (5)	0.9074 (4)	0.3524 (3)	0.0684 (13)	
H2A	0.8441	0.9889	0.3563	0.103*	
H2B	0.8855	0.8621	0.3796	0.103*	
H2C	0.7380	0.8942	0.3730	0.103*	
C3	0.7113 (5)	0.3063 (4)	0.3214 (3)	0.0656 (12)	
H3A	0.6210	0.2828	0.3188	0.079*	
H3B	0.7142	0.3909	0.3180	0.079*	
C4	0.4406 (5)	1.0546 (4)	0.0497 (3)	0.0729 (14)	
H4	0.4007	1.0658	0.0034	0.087*	
C5	0.3894 (5)	1.1102 (4)	0.1121 (3)	0.0676 (13)	
H5	0.3159	1.1567	0.1068	0.081*	
C6	0.9239 (5)	0.1282 (4)	0.0326 (3)	0.0688 (13)	
H6	0.9285	0.1148	−0.0188	0.083*	
C7	1.0183 (5)	0.0806 (4)	0.0793 (3)	0.0681 (13)	
H7	1.0848	0.0364	0.0582	0.082*	
C8	0.5478 (5)	0.9837 (4)	0.0534 (3)	0.0648 (12)	
H8	0.5806	0.9463	0.0111	0.078*	
C9	0.8237 (4)	0.1949 (4)	0.0606 (2)	0.0586 (11)	
H9	0.7607	0.2275	0.0296	0.070*	
C10	0.8240 (4)	0.8715 (4)	0.2708 (2)	0.0567 (11)	
H10A	0.9077	0.8915	0.2494	0.068*	
H10B	0.8145	0.7872	0.2679	0.068*	
C11	1.0161 (4)	0.0968 (4)	0.1559 (3)	0.0586 (11)	
H11	1.0800	0.0649	0.1866	0.070*	
C12	0.7925 (4)	0.8299 (3)	0.1041 (2)	0.0528 (10)	
H12A	0.8825	0.8395	0.1190	0.063*	
H12B	0.7856	0.8511	0.0515	0.063*	
C13	0.6274 (4)	0.6663 (3)	0.1021 (2)	0.0473 (9)	
H13	0.5662	0.7196	0.0847	0.057*	
C14	0.8429 (4)	0.6215 (3)	0.1381 (2)	0.0494 (10)	
H14	0.9287	0.6438	0.1459	0.059*	
C15	0.5904 (4)	0.5521 (3)	0.1160 (2)	0.0463 (9)	
H15	0.5044	0.5301	0.1085	0.056*	
C16	0.4456 (4)	1.0978 (3)	0.1817 (3)	0.0551 (10)	
H16	0.4118	1.1361	0.2234	0.066*	
C17	0.6048 (4)	0.9707 (3)	0.1238 (2)	0.0481 (10)	
C18	0.7536 (4)	0.7026 (3)	0.1137 (2)	0.0429 (9)	
C19	0.8053 (4)	0.5061 (3)	0.1511 (2)	0.0479 (9)	
H19	0.8670	0.4522	0.1671	0.058*	
N4	0.6289 (3)	0.9968 (3)	0.25067 (19)	0.0487 (8)	
C20	0.8219 (4)	0.2108 (3)	0.1384 (2)	0.0438 (9)	
C21	0.7811 (4)	0.2564 (3)	0.2563 (2)	0.0450 (9)	
C22	0.6790 (4)	0.4702 (3)	0.1407 (2)	0.0409 (8)	
C23	0.9151 (4)	0.1625 (3)	0.1859 (2)	0.0460 (9)	
C24	0.5549 (4)	1.0261 (3)	0.1880 (2)	0.0462 (9)	
N3	0.7116 (3)	0.9081 (3)	0.14849 (19)	0.0469 (8)	
C25	0.7199 (4)	0.9271 (3)	0.2246 (2)	0.0462 (9)	
N1	0.8875 (3)	0.1927 (3)	0.25983 (19)	0.0512 (8)	
N2	0.7370 (3)	0.2705 (2)	0.18419 (18)	0.0431 (7)	
C26	0.6341 (4)	0.3469 (3)	0.1584 (2)	0.0476 (9)	
H26A	0.5673	0.3506	0.1966	0.057*	
H26B	0.5955	0.3134	0.1136	0.057*	

### Atomic displacement parameters (Å^2^)

**Table:**

	*U*^11^	*U*^22^	*U*^33^	*U*^12^	*U*^13^	*U*^23^
C1	0.117 (5)	0.110 (4)	0.052 (3)	0.041 (4)	0.011 (3)	−0.003 (3)
C2	0.081 (3)	0.068 (3)	0.056 (3)	0.009 (3)	−0.011 (3)	0.004 (2)
C3	0.067 (3)	0.069 (3)	0.061 (3)	0.018 (2)	0.008 (2)	−0.004 (2)
C4	0.090 (4)	0.064 (3)	0.064 (3)	0.000 (3)	−0.021 (3)	0.010 (3)
C5	0.062 (3)	0.056 (3)	0.085 (4)	0.000 (2)	−0.015 (3)	0.014 (3)
C6	0.084 (4)	0.065 (3)	0.058 (3)	−0.004 (3)	0.016 (3)	−0.004 (2)
C7	0.068 (3)	0.059 (3)	0.078 (3)	0.003 (2)	0.020 (3)	−0.011 (2)
C8	0.084 (3)	0.052 (2)	0.058 (3)	0.002 (2)	−0.007 (3)	0.003 (2)
C9	0.060 (3)	0.057 (2)	0.058 (3)	−0.008 (2)	0.000 (2)	0.003 (2)
C10	0.058 (3)	0.054 (2)	0.058 (3)	0.009 (2)	−0.005 (2)	−0.001 (2)
C11	0.049 (2)	0.052 (2)	0.074 (3)	0.005 (2)	0.004 (2)	−0.004 (2)
C12	0.065 (3)	0.037 (2)	0.056 (2)	−0.0053 (19)	0.012 (2)	0.0022 (18)
C13	0.051 (2)	0.041 (2)	0.051 (2)	0.0031 (18)	−0.0063 (19)	0.0059 (17)
C14	0.041 (2)	0.042 (2)	0.065 (3)	−0.0067 (17)	0.0014 (19)	−0.0016 (18)
C15	0.041 (2)	0.044 (2)	0.054 (2)	−0.0034 (17)	−0.0085 (18)	−0.0004 (18)
C16	0.051 (2)	0.044 (2)	0.070 (3)	−0.0003 (19)	−0.001 (2)	0.007 (2)
C17	0.060 (3)	0.0322 (18)	0.052 (2)	−0.0064 (18)	0.000 (2)	0.0065 (16)
C18	0.052 (2)	0.0358 (18)	0.041 (2)	−0.0026 (17)	0.0066 (18)	−0.0046 (15)
C19	0.042 (2)	0.0382 (19)	0.063 (2)	0.0003 (17)	−0.0072 (19)	0.0028 (18)
N4	0.0480 (18)	0.0435 (17)	0.0546 (19)	−0.0018 (15)	0.0026 (16)	−0.0025 (15)
C20	0.050 (2)	0.0317 (17)	0.049 (2)	−0.0045 (16)	0.0024 (19)	0.0028 (16)
C21	0.048 (2)	0.041 (2)	0.045 (2)	−0.0006 (18)	0.0005 (17)	0.0027 (17)
C22	0.044 (2)	0.0409 (19)	0.0380 (18)	0.0006 (16)	−0.0033 (16)	−0.0016 (15)
C23	0.044 (2)	0.0373 (19)	0.057 (2)	−0.0043 (16)	0.0030 (19)	−0.0010 (17)
C24	0.049 (2)	0.0367 (18)	0.054 (2)	−0.0081 (17)	−0.0017 (19)	0.0057 (17)
N3	0.056 (2)	0.0364 (16)	0.0484 (18)	0.0004 (15)	0.0037 (16)	0.0001 (14)
C25	0.051 (2)	0.039 (2)	0.049 (2)	−0.0043 (18)	0.0016 (18)	0.0004 (16)
N1	0.0480 (19)	0.0485 (19)	0.057 (2)	0.0046 (16)	0.0009 (16)	−0.0002 (16)
N2	0.0432 (17)	0.0356 (15)	0.0504 (18)	0.0028 (13)	−0.0008 (15)	0.0022 (14)
C26	0.045 (2)	0.0358 (18)	0.062 (2)	0.0002 (17)	−0.0034 (19)	0.0048 (17)

### Geometric parameters (Å, °)

**Table:**

C1—C3	1.492 (6)	C12—N3	1.456 (5)
C1—H1A	0.9600	C12—C18	1.520 (5)
C1—H1B	0.9600	C12—H12A	0.9700
C1—H1C	0.9600	C12—H12B	0.9700
C2—C10	1.510 (6)	C13—C18	1.380 (5)
C2—H2A	0.9600	C13—C15	1.383 (5)
C2—H2B	0.9600	C13—H13	0.9300
C2—H2C	0.9600	C14—C18	1.376 (5)
C3—C21	1.479 (5)	C14—C19	1.395 (5)
C3—H3A	0.9700	C14—H14	0.9300
C3—H3B	0.9700	C15—C22	1.380 (5)
C4—C8	1.371 (7)	C15—H15	0.9300
C4—C5	1.384 (7)	C16—C24	1.397 (5)
C4—H4	0.9300	C16—H16	0.9300
C5—C16	1.376 (6)	C17—N3	1.384 (5)
C5—H5	0.9300	C17—C24	1.404 (5)
C6—C9	1.377 (6)	C19—C22	1.377 (5)
C6—C7	1.391 (7)	C19—H19	0.9300
C6—H6	0.9300	N4—C25	1.316 (5)
C7—C11	1.378 (6)	N4—C24	1.393 (5)
C7—H7	0.9300	C20—N2	1.377 (5)
C8—C17	1.393 (6)	C20—C23	1.394 (5)
C8—H8	0.9300	C21—N1	1.317 (5)
C9—C20	1.396 (6)	C21—N2	1.372 (5)
C9—H9	0.9300	C22—C26	1.516 (5)
C10—C25	1.495 (5)	C23—N1	1.390 (5)
C10—H10A	0.9700	N3—C25	1.375 (5)
C10—H10B	0.9700	N2—C26	1.449 (4)
C11—C23	1.390 (5)	C26—H26A	0.9700
C11—H11	0.9300	C26—H26B	0.9700
			
C3—C1—H1A	109.5	C18—C13—H13	119.5
C3—C1—H1B	109.5	C15—C13—H13	119.5
H1A—C1—H1B	109.5	C18—C14—C19	120.3 (4)
C3—C1—H1C	109.5	C18—C14—H14	119.8
H1A—C1—H1C	109.5	C19—C14—H14	119.8
H1B—C1—H1C	109.5	C13—C15—C22	121.1 (4)
C10—C2—H2A	109.5	C13—C15—H15	119.5
C10—C2—H2B	109.5	C22—C15—H15	119.5
H2A—C2—H2B	109.5	C5—C16—C24	118.1 (4)
C10—C2—H2C	109.5	C5—C16—H16	120.9
H2A—C2—H2C	109.5	C24—C16—H16	120.9
H2B—C2—H2C	109.5	N3—C17—C8	132.6 (4)
C21—C3—C1	114.1 (4)	N3—C17—C24	105.4 (3)
C21—C3—H3A	108.7	C8—C17—C24	122.0 (4)
C1—C3—H3A	108.7	C13—C18—C14	118.3 (3)
C21—C3—H3B	108.7	C13—C18—C12	121.3 (4)
C1—C3—H3B	108.7	C14—C18—C12	120.3 (4)
H3A—C3—H3B	107.6	C22—C19—C14	121.5 (4)
C8—C4—C5	122.7 (5)	C22—C19—H19	119.3
C8—C4—H4	118.6	C14—C19—H19	119.3
C5—C4—H4	118.6	C25—N4—C24	104.6 (3)
C16—C5—C4	121.0 (5)	N2—C20—C9	131.3 (4)
C16—C5—H5	119.5	N2—C20—C23	105.9 (3)
C4—C5—H5	119.5	C9—C20—C23	122.8 (4)
C9—C6—C7	121.6 (4)	N1—C21—N2	112.6 (3)
C9—C6—H6	119.2	N1—C21—C3	125.5 (4)
C7—C6—H6	119.2	N2—C21—C3	121.9 (3)
C11—C7—C6	121.9 (4)	C19—C22—C15	117.7 (3)
C11—C7—H7	119.1	C19—C22—C26	122.5 (3)
C6—C7—H7	119.1	C15—C22—C26	119.7 (3)
C4—C8—C17	116.5 (5)	C11—C23—N1	130.6 (4)
C4—C8—H8	121.8	C11—C23—C20	119.8 (4)
C17—C8—H8	121.8	N1—C23—C20	109.6 (3)
C6—C9—C20	116.2 (4)	N4—C24—C16	130.3 (4)
C6—C9—H9	121.9	N4—C24—C17	110.1 (3)
C20—C9—H9	121.9	C16—C24—C17	119.6 (4)
C25—C10—C2	114.1 (4)	C25—N3—C17	106.3 (3)
C25—C10—H10A	108.7	C25—N3—C12	126.6 (4)
C2—C10—H10A	108.7	C17—N3—C12	126.9 (3)
C25—C10—H10B	108.7	N4—C25—N3	113.5 (4)
C2—C10—H10B	108.7	N4—C25—C10	125.1 (4)
H10A—C10—H10B	107.6	N3—C25—C10	121.4 (4)
C7—C11—C23	117.8 (4)	C21—N1—C23	105.2 (3)
C7—C11—H11	121.1	C21—N2—C20	106.6 (3)
C23—C11—H11	121.1	C21—N2—C26	127.6 (3)
N3—C12—C18	112.1 (3)	C20—N2—C26	125.1 (3)
N3—C12—H12A	109.2	N2—C26—C22	113.8 (3)
C18—C12—H12A	109.2	N2—C26—H26A	108.8
N3—C12—H12B	109.2	C22—C26—H26A	108.8
C18—C12—H12B	109.2	N2—C26—H26B	108.8
H12A—C12—H12B	107.9	C22—C26—H26B	108.8
C18—C13—C15	121.1 (4)	H26A—C26—H26B	107.7
			
C8—C4—C5—C16	0.8 (7)	N3—C17—C24—N4	−0.6 (4)
C9—C6—C7—C11	0.4 (7)	C8—C17—C24—N4	179.1 (4)
C5—C4—C8—C17	−0.6 (7)	N3—C17—C24—C16	179.5 (3)
C7—C6—C9—C20	−0.5 (7)	C8—C17—C24—C16	−0.8 (6)
C6—C7—C11—C23	0.4 (7)	C8—C17—N3—C25	−179.0 (4)
C18—C13—C15—C22	−1.1 (6)	C24—C17—N3—C25	0.7 (4)
C4—C5—C16—C24	−0.9 (6)	C8—C17—N3—C12	−3.7 (7)
C4—C8—C17—N3	−179.8 (4)	C24—C17—N3—C12	176.0 (3)
C4—C8—C17—C24	0.6 (6)	C18—C12—N3—C25	77.0 (5)
C15—C13—C18—C14	1.3 (6)	C18—C12—N3—C17	−97.5 (4)
C15—C13—C18—C12	−175.4 (4)	C24—N4—C25—N3	0.1 (4)
C19—C14—C18—C13	−0.5 (6)	C24—N4—C25—C10	−179.7 (4)
C19—C14—C18—C12	176.3 (4)	C17—N3—C25—N4	−0.5 (4)
N3—C12—C18—C13	53.7 (5)	C12—N3—C25—N4	−175.9 (3)
N3—C12—C18—C14	−123.0 (4)	C17—N3—C25—C10	179.3 (3)
C18—C14—C19—C22	−0.5 (6)	C12—N3—C25—C10	3.9 (6)
C6—C9—C20—N2	−179.5 (4)	C2—C10—C25—N4	−3.5 (6)
C6—C9—C20—C23	−0.2 (6)	C2—C10—C25—N3	176.8 (4)
C1—C3—C21—N1	−5.2 (7)	N2—C21—N1—C23	−0.3 (4)
C1—C3—C21—N2	173.1 (4)	C3—C21—N1—C23	178.1 (4)
C14—C19—C22—C15	0.8 (6)	C11—C23—N1—C21	179.6 (4)
C14—C19—C22—C26	−176.7 (4)	C20—C23—N1—C21	0.3 (4)
C13—C15—C22—C19	0.0 (6)	N1—C21—N2—C20	0.2 (4)
C13—C15—C22—C26	177.6 (4)	C3—C21—N2—C20	−178.3 (4)
C7—C11—C23—N1	179.7 (4)	N1—C21—N2—C26	−171.1 (3)
C7—C11—C23—C20	−1.1 (6)	C3—C21—N2—C26	10.4 (6)
N2—C20—C23—C11	−179.6 (3)	C9—C20—N2—C21	179.4 (4)
C9—C20—C23—C11	1.0 (6)	C23—C20—N2—C21	0.0 (4)
N2—C20—C23—N1	−0.2 (4)	C9—C20—N2—C26	−9.0 (6)
C9—C20—C23—N1	−179.7 (4)	C23—C20—N2—C26	171.6 (3)
C25—N4—C24—C16	−179.8 (4)	C21—N2—C26—C22	86.5 (5)
C25—N4—C24—C17	0.3 (4)	C20—N2—C26—C22	−83.2 (4)
C5—C16—C24—N4	−178.9 (4)	C19—C22—C26—N2	−2.2 (6)
C5—C16—C24—C17	0.9 (5)	C15—C22—C26—N2	−179.6 (3)
